# Clinical relevance of circulating angiogenic cells in patients with ischemic stroke

**DOI:** 10.1186/s12872-021-02421-8

**Published:** 2022-03-21

**Authors:** Francisco Rodríguez-Esparragón, Laura B. Torres-Mata, Juan Carlos López-Fernández, Laura Cappiello, Jesús M. González-Martín, Bernardino Clavo, Jaime A. Serna-Gómez, Lidia Estupiñán-Quintana, Cristina Torres-Ascensión, Jesús Villar

**Affiliations:** 1 Research Unit, Hospital Universitario de Gran Canaria Dr. Negrín, Barranco de La Ballena S/N, 35019 Las Palmas de Gran Canaria, Spain; 2 Department of Neurology, Hospital Universitario Dr. Negrín, Las Palmas de Gran Canaria, Spain; 3 Chronic Pain Unit, Hospital Universitario Dr. Negrín, Las Palmas de Gran Canaria, Spain; 4 Radiation Oncology Department, Hospital Universitario Dr. Negrín, Las Palmas de Gran Canaria, Spain; 5 Universitary Institute for Research in Biomedicine and Health (iUIBS), Molecular and Translational Pharmacology Group, University of Las Palmas de Gran Canaria, Las Palmas de Gran Canaria, Spain; 6 Spanish Group of Clinical Research in Radiation Oncology (GICOR), 28029 Madrid, Spain; 7 Research Network On Health Services in Chronic Diseases (REDISSEC), Instituto de Salud Carlos III, Madrid, Spain; 8 Department of Cardiovascular Surgery, Hospital Universitario Dr. Negrín, Las Palmas de Gran Canaria, Spain; 9 CIBER de Enfermedades Respiratorias, Instituto de Salud Carlos III, Madrid, Spain; 10 Li Ka Shing Knowledge Institute at St Michael’s Hospital, Toronto, ON Canada

**Keywords:** Ischemic stroke, Endothelial progenitor cells, Circulating angiogenic cells, Colony forming cells, Late outgrowth endothelial cells, Biomarkers, Gene, Functionality

## Abstract

**Background:**

Endothelial progenitor cells (EPCs) are circulating angiogenic cells with endothelial features associated with risk for stroke. We aimed to delve into their functional characteristics. EPCs were isolated and cultured from Ischemic Stroke (IS) patients and predictors of their variance evaluated.

**Methods:**

This is a single-center observational study evaluating 187 consecutively hospitalized patients with IS. EPCs were isolated from blood samples. The number of circulating angiogenic cells (CACs), colony-forming units (CFU-ECs) and the emergence of late outgrowths endothelial cells (LOECs) were counted. We collected clinical variables and measured the stromal cell-derived factor 1 alpha (SDF1α) serum levels. We also examined the relative telomere length and the expression of osteogenic gene markers in CACs.

**Results:**

CACs counts and CFU-ECs colony numbers were positively correlated (rho = 0.41, *p* < 0.001, n = 187). We found significant differences according to whether thrombolytic treatment was performed in the distribution of CFU-ECs (odds ratio (OR) = 2.5; 95% confidence interval (CI) 1.01–6.35; *p* = 0.042) and CACs (OR = 4.45; 95% IC 1.2–15.5; *p* = 0.012). The main determinants of CACs variation were the number of risks factors, thrombolysis treatment, arterial hypertension, LOECs occurrence, and the vascular endothelial growth factor expression, whereas CFU-ECs variations depended on hemoglobin content and the relative reduction in the National Institutes of Health Stroke Scale (NIHSS) criteria. The main predictors of LOECs appearance were thrombolysis and length of hospital stay.

**Conclusions:**

Our study supports the relevance of patient risk factors and treatments in the analysis of the functional properties of EPCs.

## Background

Ischemic stroke (IS) represents 80–85% of stroke subtypes and it is one of the leading causes of human death and disability worldwide [1, 2]. The formation of new blood vessels or vasculogenesis substantially contribute to the neurovascular repair process after an IS [3]. Different populations of progenitor cells attracted by different trophic stimuli have been identified in remodeling areas surrounding ischemic brain areas [4–6]. Asahara et al., [7] first reported a subset of CD34 + cells with endothelial features that were identified as endothelial progenitor cells (EPCs). Soon after, it was identified that when culturing mononuclear cells (MNCs) from human peripheral blood samples, two types of cells can be differentiated to the so called early EPCs and late EPCs [4, 5].

Further studies identified early EPCs as pro-angiogenic hematopoietic cells [5] which are thought to be the first cells to invade a site in response to an angiogenic stimuli and to guide resident and circulating endothelial cells and late EPCs to the injured sites [8]. Unlike early EPCs, late EPCs have the ability to form capillary-like tubes and possess high growth potential [5, 9]. Early EPCs has been more accurately defined as circulating angiogenic cells (CACs) [10]. Both types of cells can be distinguished by using fluorochrome-labeled antibodies and fluorescence activated cell analysis or by using different culture conditions [5, 11–13]. CACs arise from peripheral blood cells seeded on fibronectin-coated dishes [11]. Nevertheless, by using some culture features it can be obtained clusters of cells referred to as colony forming units (CFU-ECs) that displays cells with spindle-shaped morphology and emerge following 4–9 days of culture [14]. Both myeloid progenitor and lymphoid cells participate in the formation of these colonies [4, 15], and it has been reported an inverse association of EPCs-related phenotypes with cardiovascular risk factors and aggregate cardiovascular risk [11, 14, 16, 17].

We aimed to evaluate functional characteristic of early CACs and CFU-ECs counts and late EPCs occurrence in IS patients. We use a modified Rankin scale score [18] and the National institute of Health Stroke Scale (NIHSS) score [19] to assess clinical outcome and disease severity after disease onset.

## Methods

### Study population

This study was performed in line with the principles of the Declaration of Helsinki. Approval was granted by the Ethics Committee of the Hospital Universitario de Gran Canaria Dr. Negrín (approval 140157). Both verbal and written informed consent was obtained from all individual donors. Patients were admitted into the Stroke Unit at the Department of Neurology of the Hospital Universitario de Gran Canaria Doctor Negrín, Las Palmas de Gran Canaria, Gran Canaria, Spain, between 2018 and 2019. After excluding other etiologies, all diagnoses were confirmed by clinical criteria as being affected by neurological deficits lasting longer than 24 h or clinical transient ischemic attacks (TIAs) in which cerebral computed tomography (CT) or magnetic resonance (MR) showed acute arterial cerebral infarction related to clinical findings. Patients with brain hematomas were also excluded. We screened 187 consecutive, adult, unrelated patients with IS, diagnosed according to the TOAST trial criteria [20]. The severity of the neurological deficit was assessed using the National Institutes of Health Stroke Scale (NIHSS) score [19] on admission (Basal-NIHSS) and at 7 and 12 days. The delta NIHSS was calculated as the difference between Basal-NIHSS and NIHSS at day 7. The relative score reduction (RR-NIHSS; delta NIHSS/B-NIHSS) and the major neurological improvement (MNI; NIHSS improvement of 0–1 or ≥ 8 at 7 days) were also calculated [21]. A favorable outcome was defined as a score 0–2 on the Rankin scale score [18]. Patients were considered eligible for recombinant tissue plasminogen activator (rt‐PA) treatment if they fulfill the inclusion and exclusion criteria as stated in the European Cooperative Acute Stroke Study (ECASS III) [22]. Patients with ischemic stroke caused by a proximal large artery occlusion in the anterior circulation that were candidates for intra-arterial mechanical thrombectomy were transferred to a stroke center with expertise in the use of stent retrievers for acute ischemic stroke.

### Clinical data collection

In all patients, we recorded the following data: demographics (age and sex); presence of traditional vascular risk factors, including previously or de novo diagnosed arterial hypertension, diabetes mellitus, hypercholesterolemia, coronary artery disease, smoking habit, alcohol abuse, peripheral artery disease, atrial fibrillation, previous transient ischemic attack, previous cerebral infarct, antihypertensive treatment, treatment with statins before the onset of stroke, body mass index (BMI), number of days after admission, thrombolysis treatment, thrombectomy treatment, and levels of glucose, HbA1c, urate, creatinine, albumin, total proteins, folic acid, vitamin B12 and homocysteine, T4, TSH. A complete lipid profile and detailed blood counts were also measured.

### Cells cultures and immunofluorescent staining

Peripheral blood was sampled from patients 7 to 9 days after symptom onset. CACs were cultured and counted as described elsewhere [11, 12]. Cultured CACs on days 7 and 10 were manually counted using an inverted microscope. CACs numbers were analyzed for each patient as the mean of four randomly chosen fields per well of six multiwells. CFU-EC assay was performed as described elsewhere [23]. Colonies were counted on days 7 and 10. LOECs were obtained seeding a similar number of MNCs on a 35 mm plated coated with collagen containing EBM-2 supplemented medium for up of two weeks.

CACs and late EPC colonies were prepared for lecithin binding and uptake studies as previously reported [24].

### Immunophenotype and gene expression in CACs and LOECs

Gene expression patterns of hematopoietic (CD45), steaminess (CD133) and endothelial (KDR, VEGF and CD31) markers were evaluated by retro-transcription and real-time polymerase chain reaction in CACs and LOECs. The relative expression of CXC-motif ligand 12 and CXC chemokine receptor 7, 4 and (CXCL12, CXCR7, CXCR4) and osteogenic gene markers osteopontin (OPN, vSSP-1, BNSP, bone sialoprotein), osteocalcin (OC, BGLAP) and osteonectin (ON, Secreted Protein, Acidic, Cysteine-Rich, SPARC) were analyzed in CACs. The identity of the PCR products was confirmed by melting analysis and size determination in agarose gels. Gene expression data were normalized to GAPDH expression level to obtain ΔC_T_ (cycle thresholds) and positive controls were used as calibrators. Calibrators were constructed with cDNAs synthesized from RNAs isolated of different cell sources known to express each analyzed gene.

### Relative telomere lengths

Telomere mass in isolated peripheral blood MCNs and EPCs subpopulations (CACs and LOECs) was measured as described [25].

### Biochemical determinations

Biochemical determinations were performed within usual clinical routine protocols. Stromal cell-derived factor 1α (SDF1α) was measured in the plasma of analyzed patients using a R&D System ELISA kit.

### Statistical analysis

Data were analyzed using the R Software [26] and reported as mean ± standard deviation (SD) or frequencies and percentages. Continuous variables were tested for normal distribution using the Kolmogorov–Smirnov test. Comparisons between groups were analyzed using the 2-tailed unpaired Student t test or analysis of variance for normally distributed variables and using the Kruskal–Wallis test for non-normally distributed variables. Due to the great number of observations with no cell counts, CACs and CFU-ECs counts were also transformed using the formula, ln(x + x/2) to reduce the skewness of the data. Univariate correlations between quantitative variables and CACs and CFU-ECs counts were performed with normalized log-transformed CACs and CFU-ECs counts and tested for normality. Cell-counts were coded as dummy variables presence/absence and the distributions were analyzed according to the clinical categorical variables recorded. Categorical variables were compared using the Pearson χ^2^ test. Odds ratios (OR) and 95% confidence intervals (CI) were calculated. To identify independent predictors of CACs counts and CFU-ECs colony counts, stepwise linear regression analyses were performed computing both forward stepwise and backward selection to choose an optimal simple model without compromising accuracy. To identify predictors of LOEC appearance logistic regression analyses were done. A value of *p* < 0.05 was considered statistically significant.

## Results

### Study population

Baseline characteristics of the 187 study subjects are summarized in Table 1. After adjusting for all inclusion criteria, 25% of the patients were found to be potentially eligible and were treated with intravenous thrombolysis. Only 1 patient treated with mechanic thrombectomy was finally included.

**Table 1:** Clinical patient characteristics

Characteristic	
Median, range, sample size	
Age	72, 31–103, 187
Basal NIHSS	7.28, 0–25, 139
NIHSS (7 day)	3.17, 0–24, 115
Percentage, sample size	
Sex (woman)	33.6%, 49
IV rtPA	24.7%, 36
Hypertension	82.6%, 123
Diabetes mellitus	42.3%, 63
Hyperlipidemia	66.9%, 99
Current Smoking	25.9%, 38
Alcohol	12.9%, 19
Stroke etiology (TOAST)	
Atheromatous	12.9%, 24
Cardioembolic	35.2%, 65
Lacunar	9.7%, 18
Infrequent etiology	3.2%, 6
Undetermined etiology	39%, 72

Basic demographic and clinical patient characteristics (NIHSS National Institute of Health Stroke Scale, IV rtPA intravenous recombinant tissue plasminogen activator, TOAST Trial of Org 10172 in Acute Stroke Treatment

### EPC ex vivo cultures

**Fig. 1:**
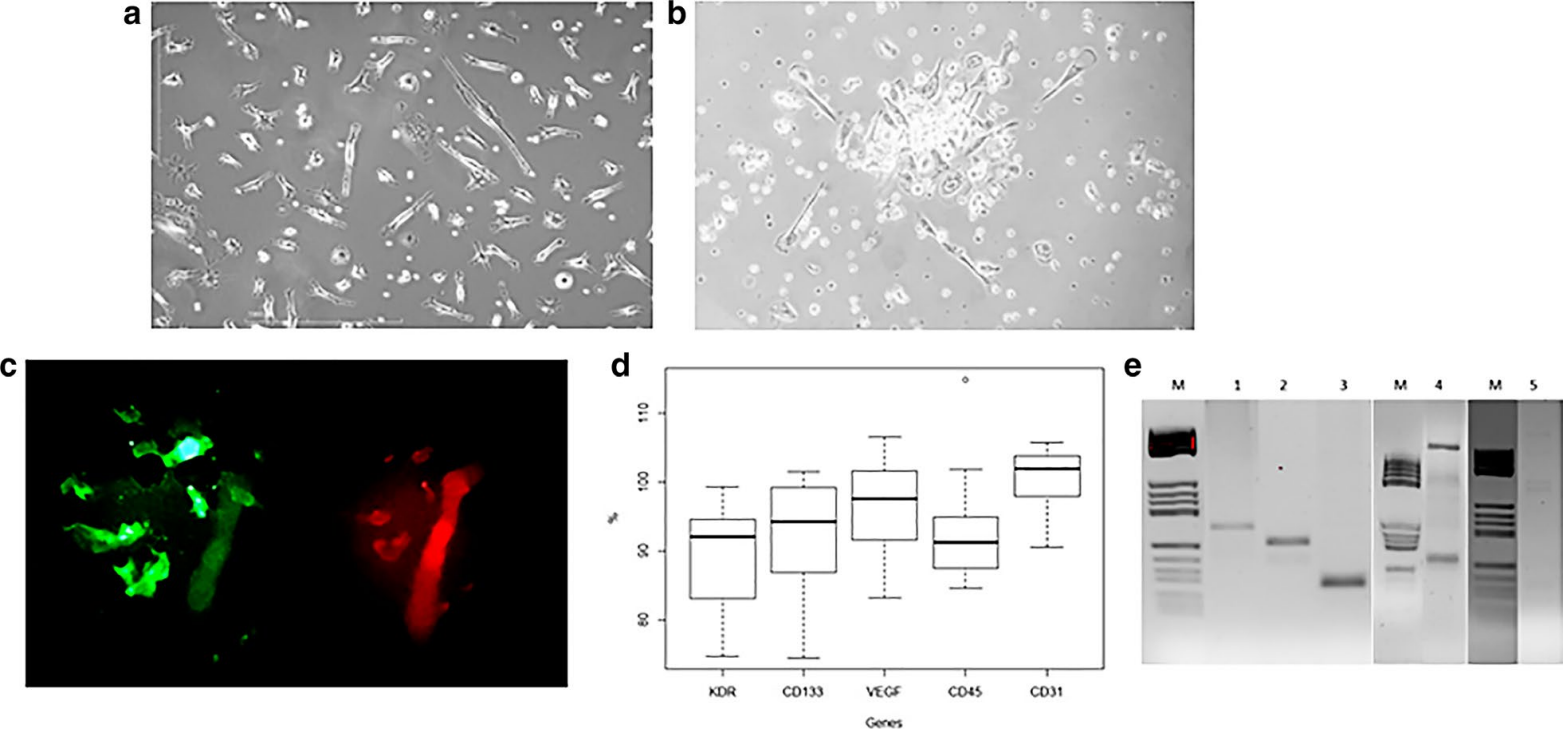
Endothelial progenitor cells (EPCs). **a** Circulating angiogenic cells (CACs) constitute a cell population enriched in monocytes that exert their angiogenic effects via paracrine. **b** Colony-forming units (CFU-ECs) are EPCs that can be obtained under certain culture conditions formed by a combination of monocytes and T cells. CFU-ECs express endothelial markers but also myeloid and hematopoietic markers. **c** CACs cultured from patients were positive for Dil-Ac-LDL uptake (red areas) and FITC-UEA-I binding (green areas). **d** Box plot displaying the distribution if the CACs analyzed marker genes. **e** Agarose gel conformation of PCR amplicons sixes: (1) VEGF, (2) KDR, (3) CD31, (4) CD45 and CD133

**Fig. 2:**
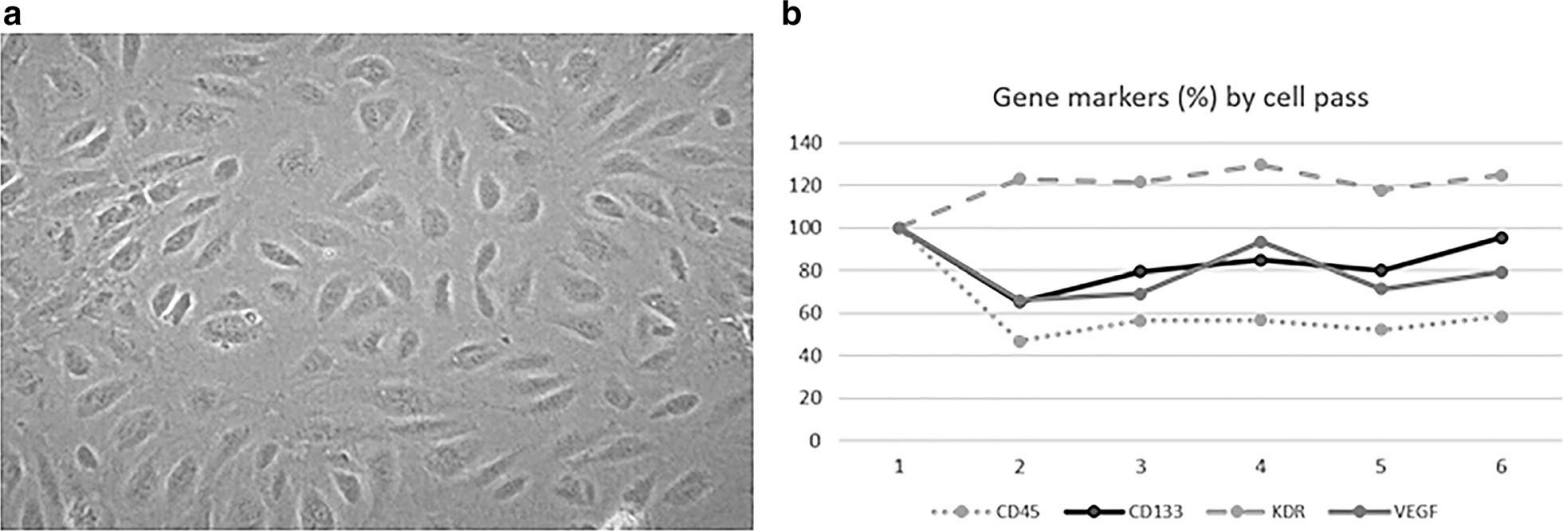
Endothelial progenitor cells (EPCs). **a** Late outgrowth EPCs also referred to as late EPCs, blood outgrowth endothelial cells, and endothelial outgrowth cells, are the only EPC subset faithfully belonging to the endothelial lineage. **b** Linear plot displaying the distribution of analyzed marker genes according cell passages

CACs counts were obtained in 51.7% of the analyzed patients (Fig. 1a). CFU-ECs were obtained in 9.7% of patients (Fig. 1b). LOECs were obtained in 13.4% of analyzed samples (Fig. 2a).

### Immunophenotypes and gene expression in CACs

We found similar percentages of CACs expressing CD45 and CD133 than CACs expressing only CD45. CACs were positive for KDR, VEGF and CD31 (Fig. 1d, e). Gene expression pattern on LOECs are depicted in Fig. 2b.

**Fig. 3:**
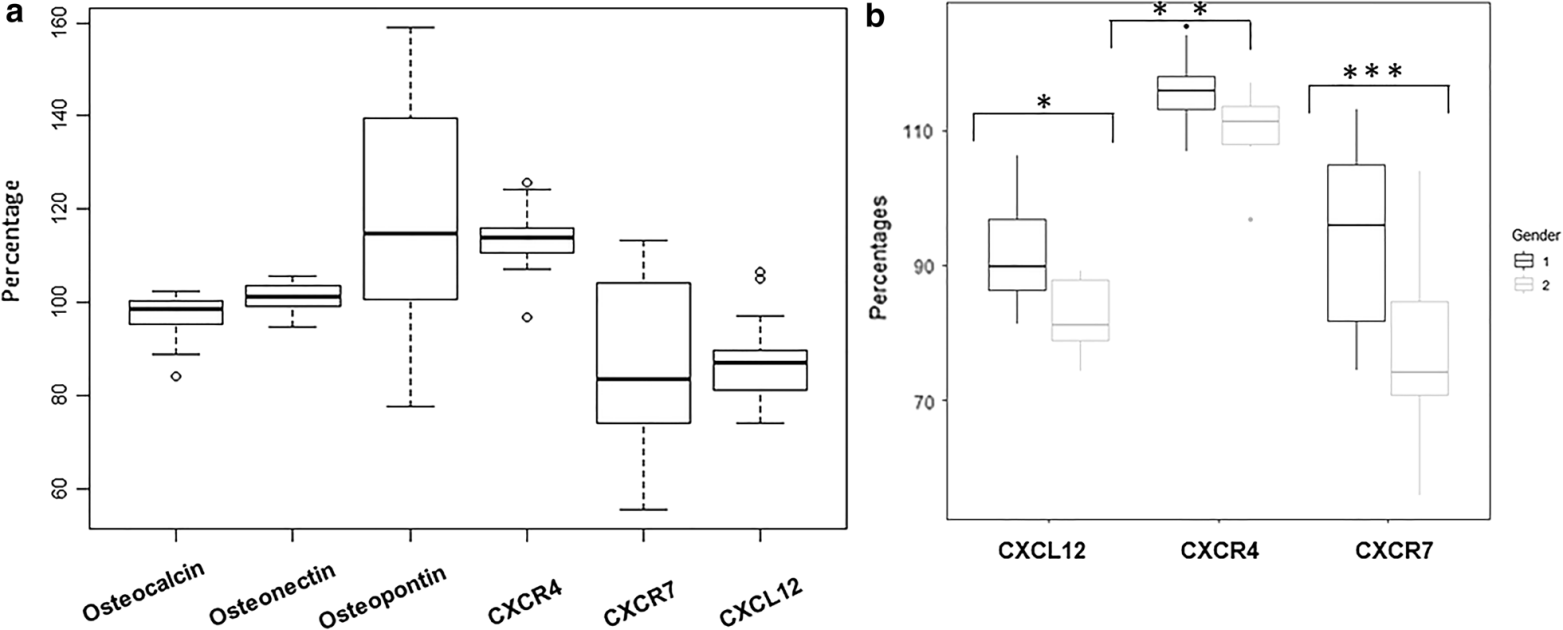
**a** Box plot showing the mean analyzed osteogenic and CXCL12/CXCR4/CXCR7 chemokine axis gene markers expression in CACs. Whiskers and box bands denote quartiles. **b** Box plot showing gene expression between male and female. Comparison using Mann–Whitney U-test for independent samples showed significant differences (*p = 0.03, **p = 0.006, ***p = 0.009)

We observed a discrete expression pattern for OC, OPN and ON genes (Fig. 3a). All evaluated cells expressed OC and ON with a very small deviation with respect to the reference value. A 72.7% of analyzed CACs expressed OPN and correlated with the numbers of CACs (rho = 0.571, *p* = 0.017). We also observed a discrete pattern of expression of the CXCL12/CXCR4/CXCR7 chemokine axis genes in CACs (Fig. 3a). CXCL12 and CXCR4 expression was strongly correlated (rho = 0.821; *p* < 0.001). Only CXCR7 expression correlated with SDF1α (rho = 0.542; *p* = 0.01). CXCl12, CXCR4 and CXCR7 genes expressions were significantly increased in men (Fig. 3b).

### Relative telomere lengths in blood cells and CACs and LOECs

There was a negative and non-significant correlation between age and the telomere length in MNCs; however both CACs and LOECs showed a correlation with age that reached significance for LOEC (rho = 0.89, *p* = 0.037). Blood cells TLM was negatively correlated with BMI (rho = -0.219; *p* = 0.026) and plasma SDF-1α (rho = − 0.482, *p* < 0.001) whereas no correlations were observed between TLM and CAC or CFU-EC. TLM-CACs correlated with CFU-ECs (rho = 0.567; *p* = 0.022) and HDL concentration (rho = 0.592; *p* = 0.033) and negatively with triglycerides concentration (rho = − 0.654; *p* = 0.015).

### CFU-ECs, CACs and LOECs and clinical continuous variables

There was a correlation between CACs counts and CFU-ECs numbers (rho = 0.405; *p* < 0.001). CFU-ECs correlated with NIHSS at day 7 (rho = 0.532; *p* = 0.016), hemoglobin (rho = 0.469; *p* = 0.021) and negatively with the urine albumin-creatinine index (rho = -0.567; *p* = 0.027) whereas CACs correlated with the number of previously risk factors (rho = 0.231; *p* = 0.015). CFU-ECs and CACs counts transformed to their natural logarithm were correlated with T4 level (rho = 0.283; *p* = 0.002 and rho = 0.197; *p* = 0.033).

Mean CACs was higher in patients from which LOECs were obtained with respect to those in which LOECs were undetected. When patients were grouped according to LOEC emergence, CAC was statistically different (*p* = 0.031).

### CFU-ECs, CACs and LOECs and clinical categorical variables

There were no differences in age and sex distribution among patients with respect to CACs and CFU-ECs presence/absence (Table 2). We found significant differences in the distribution of CFU-ECs appearance (OR = 2.5; 95% IC 1.01–6.35; *p* = 0.042) and CACs occurrence (OR = 4.45; 95% IC 1.2–15.5; *p* = 0.012) in relation to thrombolytic treatment. Mean CFU-EC and mean CAC were higher in patients receiving thrombolytic therapy than in non-treated patient (*p* = 0.044 and *p* = 0.001, respectively).

**Table 2:** Patient Characteristics according circulating angiogenic cells and clusters presence/absence

	CACs (+)	CACs (−)	*p* value	CFU-ECs (+)	CFU-ECs (−)	*p* value
	(n = 144)	(n = 43)		(n = 27)	(n = 160)	
Age (years)	72.5 ± 11	70.5 ± 15	0.346	74.2 ± 10	71.7 ± 13	0.331
Sex (% man)	87 (75.7)	28 (24.3)	0.613	13 (11.3)	102 (88.7)	0.114
BMI (Kg/m^2^)	32.9 ± 37	31.2 ± 10	0.825	29.11 ± 5	33.2 ± 36	0.891
Waist (cm)	101 ± 11	101 ± 4	0.835	102.5 ± 4	100.6 ± 11	0.470
*Laboratory*						
Fasting glucose (mg/dl)	119 ± 47	124 ± 46	0.662	125 ± 69	119 ± 42	0.805
Hemoglobin	13.3 ± 2	13.5 ± 5	0.541	12.7 ± 2	13.5 ± 2	0.043
HbA1b	6.68 ± 1.3	6.67 ± 1.4	0.756	6.5 ± 1	6.7 ± 1.4	0.987
Total cholesterol (mg/dl)	163.4 ± 46	157.3 ± 44	0.488	165.7 ± 46	161 ± 45	0.671
LDL cholesterol (mg/dl)	94 ± 45	90.5 ± 43	0.569	90.8 ± 44	93.6 ± 45	0.624
HDL cholesterol (mg/dl)	42.5 ± 12	44.6 ± 14	0.391	46 ± 12	42 ± 12	0.163
Triglycerides (mg/dl)	142 ± 79	128 ± 73	0.213	127 ± 55	141 ± 81	0.043
*Anamnesis*						
Basal NIHSS	7.42 ± 6.1	6.85 ± 5.4	0.878	7.54 ± 5.6	7.23 ± 6.0	0.586
NIHSS at day 7	3.11 ± 5.2	3.36 ± 5.4	0.868	2.15 ± 4.6	3.38 ± 5.3	0.302
Basal Rankin	0.54 ± 1.1	0.52 ± 1.8	0.779	0.7 ± 1.3	0.5 ± 1.1	0.698
Rankin at day 7	1.93 ± 1.9	2.07 ± 2.1	0.807	2.13 ± 2.0	1.92 ± 2.0	0.500
Hypertension (%)	86 (75.4)	28 (24.6)	0.775	20 (17.5)	94 (82.5)	0.359
Diabetes mellitus (%)	49 (77.8)	14 (22.2)	0.664	11 (17.5)	52 (82.5)	0.678
Hyperlipidemia (%)	79 (79.8)	20 (20.2)	0.183	16 (16.2)	83 (83.8)	0.980
Current smoking (%)	29 (76.3)	9 (23.7)	0.915	7 (18.4)	31 (81.6)	0.570
Alcohol (%)	14 (73.7)	5 (26.3)	0.828	3 (15.8)	16 (84.2)	0.974
*Current medication*						
RAS inhibitors (%)	36 (78.3)	10 (21.7)	0.450	8 (17.4)	38 (82.6)	0.613
Statins (%)	59 (80.8)	14 (19.2)	0.826	13 (17.8)	60 (82.2)	0.703

CACs or CFU-ECs + or − denotes presence or absence of Circulating Angiogenic Cells and Colony-forming Units respectively. Data are present as the mean value ± standard deviation or number (%) of subjects

Mean CFU-ECs and mean CACs were slightly lower in patients with NIHSS score values >  = 12 with respect to patients with score < 12. However, there were not significant differences either NIHSS basal score or after 7 days. Mean CACs was lower in patients with improvement of ≤ 3-points than in those with > 4-points (*p* = 0.029). No difference was observed in mean CFU-EC colony number.

LOECs emergence was associated with male sex (OR = 4.162; 95% CI 1.38–12.47; *p* = 0.007) and previously diagnosed hypertension (OR = 1.2; 95% CI 1.1–1.2; *p* = 0.025). There was a higher probability to detect LOECs in those patients with SDF1α levels equal or lower than 4.3 ng/ml (*p* = 0.043).

### Determinants of CACs and LOECs

Thrombolysis treatment, hypertension, number of risk factors, variation in expression of VEGF, and LOECs appearance were main determinants of CACs variation (Table 3a). CFU-ECs variations depended on hemoglobin and the relative reduction in NIHSS criteria (Table 3b). Thrombolytic treatment and hospital length of stay significantly contributed to LOECs appearance (Table 4).

**Table 3:** Linear regression analyses

	Beta	Std. Error	*p* value	OR	2.5%	97.5%
*a Multiple linear regression analysis for CACs*						
(Intercept)	6.59239	0.97212	1.59e−08	729.5	10.5	50,438
Thrombolysis	0.86720	0.15830	1.55e−06	2.38	1.194	4.745
Hypertension	0.64078	0.21456	0.00443	1.898	0.745	4.834
LOECs	− 0.65849	0.29737	0.03158	0.518	0.142	1.891
Risks	0.20930	0.08672	0.01967	1.233	0.845	1.799
VEGF	− 0.05366	0.01025	3.58e−06	0.948	0.906	0.991

	Beta	Std. Error	Pr( >|t|)	OR	2.5%	97.5%
*b Multiple regression analysis for CFU-ECs*						
(Intercept)	− 0.87925	1.23767	0.48527	0.415	0.032	5.445
Hemoglobine	0.19561	0.08806	0.03746	1.216	1.013	1.460
RR	− 1.24604	0.42851	0.00841	0.288	0.118	0.701

*RR* relative reduction NIHSS score

**Table 4:** Logistic regression analysis for LOECs emergence

	Beta	Std. Error	*p* value	OR	2.5%	97.5%
Intercept	1.170	2.754	0.671	3.222	0.016	945
Sex	− 0.133	0.962	0.889	0.875	0.113	5.503
Hospital Stay	0.416	0.164	0.011	0.659	0.455	0.872
Thrombolysis	2.317	1.001	0.020	10.148	1.604	90.102
NIHSS-basal	− 0.134	0.112	0.233	0.874	0.677	1.065
Diabetes	1.518	1.028	0.139	4.566	0.658	1.065
HDL	0.009	0.044	0.831	1.010	0.924	1.105
LDL	0.009	0.008	0.249	1.010	0.993	1.027
Tryglicerides	− 0.001	0.003	0.941	1.000	0.991	1.007
RR	− 1.538	1.095	0.160	0.215	0.021	1.628
Risks	− 0.458	0.497	0.356	0.633	0.218	1.593
TLM	− 0.022	0.018	0.222	0.977	0.936	1.011

Sex (reference 1 for man), *HDL* high-density cholesterol, *LDL* low-density cholesterol, *RR* relative reduction in NIHSS score, Risks is the sum of the number of risk factors, *TLM* relative telomere length in MNCs

## Discussion

Several studies shown that the accumulation of risk factors and the presence of coronary, cerebral, or peripheral atherosclerosis are associated with dysfunction and reduced numbers of EPCs [14, 17, 24, 27–29]. EPCs are a promising therapeutic target for IS [30, 31] although the limited availability in blood of LOECs and the limited availability and early senescence of isolated CACs in culture conditions adversely affect its application as effective therapy [31–33]. Moreover, vascular injury or disease may be associated with activation of osteogenic genes by EPCs which could promote vascular calcification rather than normal repair processes. Thus, the functional characteristics of these cells are not sufficiently explored and should be mandatory to evaluate their safety and efficacy for the treatment of cerebral ischemia.

In this study, we determined and characterized the number of EPCs in patients with IS and evaluate the influence of clinical and biochemical determinants on their variation. In isolated CAC cells we further evaluated the expression of osteogenic genetic markers. We found that major determinants of variation in the number of CACs were the thrombolytic treatment, previously diagnosed hypertension, the number of risk factors, the variation in VEGF expression, and LOECs culture appearance, whereas independent predictors of CFU-ECs were hemoglobin and the relative reduction in NIHSS criteria. Thrombolytic treatment, and hospital length of stay contributed markedly to LOECs appearance.

We observed a low number of CFU-ECs in the evaluated patients. On the other hand, the majority of CACs (89%) did not form CFU-ECs. Despite this, the number of CACs and CFU-ECs was correlated. These apparently contradictory results [34] suggest that the higher the number of CACs, the greater the probability of obtaining CFU-ECs. On the other hand, it has been described that a low number of CFU-ECs in culture is a predictive biomarker of vascular disease [35], so there is a decreased capacity to form CFU-ECs that is characteristic of a damaged vascular condition.

In addition, patients with higher CAC and CFU-EC cells counts had an increased probability of meeting the selection criteria for thrombolytic treatment. Of note, the observation that T4 levels correlated with CACs and CFU-ECs counts could have a biological plausibility since it has been previously reported that the thyroid hormones facilitates bone marrow stem cells and/or progenitor cell release into the systemic circulation [36, 37]. Vascular injury initiates the adhesion of platelets to the exposed sub-endothelium and activated platelets secrete potent chemokines that promote the adhesion of leukocytes and other circulating cells to the site of injury promoting endothelial differentiation [38]. However, an excessive platelet activation might contribute to increased thrombogenic risk [29, 38]. Conversely, other previously described correlations that modify CFU-ECs or CACs numbers were not found [17, 39].

We observed a negative correlation between telomere length and the age of the patients in MNCs. On the contrary, a positive correlation between age and telomere length was observed in CACs and LOECs, being only statistically significant for the latter [33, 40]. Also, in CACs, CXCR4 and CXCL12 gene expression strongly correlated. Surprisingly, only CXCR7 gene expression correlated with plasma levels of SDF1α. Although it is difficult to conclude from these correlations, several studies indicate that SDF-1 regulates EPC migration via CXCR4 but not through CXCR7 [41, 42]. The CXCL12 gene which is widely expressed in many tissues throughout development, codes for SDF-1. SDF-1 is primarily thought to regulate hematopoietic stem cell migration and mobilization into and out of the bone marrow [43]. CXCR4 is a specific receptor for SDF-1 [44]. SDF-1/CXCR4 interaction plays several important physiological roles and contributes to the regulation of EPCs recruitment in ischemic tissues [45]. However, it has been found that SDF-1 could bind to CXCR4 and to CXCR7 [46].

There is a known link between osteogenesis and vasculogenesis which requires a successful coordination between osteoprogenitors and blood vessels forming cells [47]. OPN is an important pro-angiogenic factor in several pathologies [48]. In our study, we observed that all analyzed CAC expressed OC and ON and only a small percentage did not express the OPN gene. Previous studies have found higher percentage of EPC expressing OCN in patients with coronary atherosclerosis compared with subjects with normal endothelial function and no structural coronary artery disease [49]. Since we did not evaluate controls, the trend towards vascular calcification in isolated cells it is not properly explored in this study.

LOECs are bone marrow-derived precursors of vascular endothelial cells that can be mobilized to injured endothelium or ischemic tissues where participate in the repair of damaged endothelium and in the neovascularization of ischemic tissues [7, 23]. As in other reports, we observed that LOEC emergence seems to constitute a probabilistic event associated with a greater number and/or viability of CACs [4, 50].

We acknowledge some limitations to our study. First, culture CACs do not entirely correspond to EPC since they include mature endothelial cells and monocyte/macrophage-derived cells assuming an endothelial phenotype in culture. Second, CFU-ECs comprise a heterogeneous population of monocytes and T cells with distinct physiological properties [51]. Third, we did not evaluated the vasculogenic function [31] and the EPC secretome [52], and these determinants should be considered prior to evaluating the therapeutic role of EPCs in patients with IS patients. Fourth, patients who received thrombolytic treatment had more severe symptoms and large size of ischemic stroke than those who did not receive thrombolysis treatment. Moreover, EPCs could be affected by stroke subtypes, stroke severity and sampling time after stroke onset. Therefore, the association between EPCs appearance and thrombolytic treatment should be taken with caution without adjusting for these variables. Fifth, due to the absence of a control group, the cause-effect relationships between variables were not assessed and the degree of relationship between them can only be established through correlation analysis.

## Conclusions

EPCs can be a useful tool for neovascularization for the future treatment of IS, although some safety issues need to be solved. The analysis of their properties and functional characteristics is necessary before their use in the treatment of ischemic diseases. Our objective was to isolate EPCs from patients with IS to accurately distinguish their number, origin, and associate their presence or absence with clinical endpoints. In summary, we found that the determinants of the variation in the number of CAC were thrombolytic treatment, previously diagnosed hypertension, the number of risk factors, the variation in VEGF expression and the appearance of the LOEC culture, while the independent predictors of CFU-EC were hemoglobin and relative reduction in NIHSS criteria. Thrombolytic treatment and hospital stay contributed to the appearance of LOEC. More prospective studies are needed to determine whether interventions that target the number or function of EPCs can play a more significant role in the treatment of ischemic stroke.

## Data Availability

The data sets used and/or analyzed during the current study are available from the corresponding author upon reasonable request.

## Abbreviations

EPCs: Endothelial progenitor cells
IS: Ischemic stroke
CACs: Circulating angiogenic cells
CFU-ECs: Colony-forming units
LOECS: Late outgrowths endothelial cells
SDF1α: Stromal cell-derived factor 1 alpha
MNCs: Mononuclear cells
NIHSS: National Institutes of Health Stroke Scale
TOAST: Trial of ORG 10,172 in acute stroke treatment
ECASS III: European cooperative acute stroke study
TLM: Relative telomere lengths
